# Differences in severity of diffuse and focal coronary stenosis between visual and quantitative assessment

**DOI:** 10.3389/fcvm.2024.1501576

**Published:** 2024-12-16

**Authors:** Xinmao Wang, Chao Song, Heng Liu, Lin Zhou, Letian Zhang

**Affiliations:** ^1^ Department of Radiology, Daping Hospital, Army Medical University, Chongqing, China; ^2^ Chongqing Clinical Research Centre of Imaging and Nuclear Medicine, Chongqing, China

**Keywords:** diffuse and focal stenosis, quantitative coronary angiography, physician visual assessment, diameter stenosis, coronary artery disease

## Abstract

**Background:**

Coronary Artery Disease (CAD) is a leading cause of mortality, with an increasing number of patients affected by coronary artery stenosis each year. Coronary angiography (CAG) is commonly employed as the definitive diagnostic tool for identifying coronary artery stenosis. Physician Visual Assessment (PVA) is often used as the primary method to determine the need for further intervention, but its subjective nature poses challenges. This study sought to evaluate the differences of severity of diffuse and focal coronary stenosis between PVA and Quantitative Coronary Angiography (QCA).

**Methods:**

293 patients with coronary artery disease (334 coronary lesions) underwent CAG and fractional flow reserve (FFR). PVA and QCA was used to quantify diameter stenosis (DS). DS ≥50% was defined as obstructive. FFR ≤0.8 was defined as myocardial ischemia.

**Results:**

The mean ± SD age of all patients was 66 ± 9 years. ΔDS between PVA and QCA was higher in diffuse lesions (16.45 ± 7.37%) than in focal lesions (14.39 ± 7.83%) (*P* = 0.04). DS_PVA_ and DS_QCA_ had linear negative correlation (r_PVA_ = −0.3182, r_QCA_ = −0.4054) with FFR in diffuse, while in focal, DS_PVA_ and DS_QCA_ had an even stronger linear negative correlation (r_PVA_ = −0.4090, r_QCA_ = −0.4861) with FFR. ROC analysis demonstrated that DS_QCA_ had better discrimination capability for myocardial ischemia (FFR ≤ 0.80) than DS_PVA_ in different of length stenosis.

**Conclusions:**

PVA was more likely to overestimate diameter stenosis in coronary arteries than QCA, and the severity of diffuse stenosis was more likely to be overestimated than that of focal stenosis.

## 1 Introduction

Coronary Artery Disease (CAD) stands as the primary cause of mortality within the cardiovascular domain (1, 2). Various risk factors such as hypertension, hyperlipidemia, hyperglycemia, and age contribute to the development of atherosclerotic lesions in the coronary arteries (3), resulting in varying degrees of stenosis or occlusion of the vessel lumen, and the narrowing of the vessel lumen can lead to myocardial ischemia, hypoxia or necrosis (4).

Coronary angiography (CAG) as the “gold standard” for the diagnosis of coronary artery stenosis, while revascularization represents the mainstay of treatment for coronary artery stenosis (5). With the rise in popularity of CAG, there's been a surge in its usage, revealing flaws in current diagnostic criteria for coronary artery disease. Physician Visual Assessment (PVA) of coronary arteries can be inconsistent, even among experienced observers. In the 1990s, the Fractional Flow Reserve (FFR) emerged to tackle the subjectivity of PVA by assessing functional stenosis of the coronary arteries. FFR, unaffected by heart rate or blood pressure, is considered the gold standard for deciding on coronary artery revascularization (6). However, in many countries, performing FFR on every patient isn't practical due to workload, technological limitations, or costs. In contrast to PVA, Quantitative Coronary Angiography (QCA) is an objective, and highly reproducible computer-assisted technique (7). It swiftly measures and calculates the degree of coronary artery narrowing (8). Lot of study prove that, in different sex or with Acute Myocardial Infarction (or not) QCA prefer more reproducible and stability than PVA (9, 10). However, we have not yet found any relevant reports on the impact of coronary stenosis length on the accuracy of PVA and QCA.

In this study, we examined the clinical characteristics of all patients and some basic details about their affected blood vessels. Patients were divided into two groups based on the length of their coronary artery narrowing: focal stenosis (<20 mm) and diffuse stenosis (≥20 mm). We then compared how well PVA and QCA matched up with FFR, which is considered the gold standard for diagnosing coronary artery narrowing. Furthermore, the diagnostic efficacy of QCA and PVA was compared between the two types of stenosis.

## 2 Method

### 2.1 Study population

The study retrospective observed patients who visited Army Medical Centre of PLA from January 2022 to October 2023. The study was approved by the review board, and all participants signed an informed consent form. A total of 340 consecutive patients with suspected or known CAD with Chronic Coronary Syndrome who underwent CAG and FFR measurement were included in this study. The patients' basic clinical information, demographics, and imaging data were obtained directly from hospital-recorded data.

The exclusion criteria were as follows: (1) Previous history of percutaneous coronary intervention (PCI) or coronary artery bypass grafting (CABG). (2) Patients with poor coronary angiographic quality, missing clinical data, and missing imaging data. A total of 47 patients were excluded: 45 patients were excluded because of a history of PCI or CABG procedures, and 2 patients were excluded because of poor image quality (Supplementary Figure S1).

### 2.2 Coronary angiogram

Angiographs (Innova3100 IQ, pathfinder, IGS5, GE) were employed to image the patient's coronary vessels, and the contrast agent was ioprostanes (370 mg I/ml, BAYER). The surgical staff had received the relevant standardised training and completed the operation in accordance with the norms and guidelines for coronary artery disease diagnosis and treatment. In order to achieve a more effective coronary angiography, the coronary arteries were visualised by manually pushing the contrast agent, with a field of view of 160 mm × 160 mm and an imaging parameter of 15 frames/s. Following the imaging process, the images were transferred to the Cardiac x-ray Analysis 1.6 post-processing workstation for angiographic measurement.

### 2.3 Fractional flow reserve

Prior to measurement, ex vivo zero calibration of Pa and Pd was performed, followed by synchronisation of them to eliminate small errors between Pa and Pd (EQ calibration). The pressure receptors were placed as distal as possible to the coronary arteries to avoid missing lesions, and 200 ug of nitroglycerin was injected into the coronary arteries after the pressure guidewire was in place to avoid measurement errors caused by coronary spasm. Once the pressure curve had reached a steady state, an appropriate quantity of adenosine triphosphate (ATP) was injected, and the mean values of Pa and Pd were recorded in the maximally congested state in order to calculate the FFR.

### 2.4 Quantitative coronary angiography

The severity of stenosis of the diseased vessels was quantified using QCA (Cardiac x-ray Analysis 1.6 Ext.4, GE) software. The measurement procedure was as follows: firstly, the diameter of the 5F catheter was measured using the Catheter Calibration system in the software in order to ensure that the precision was within 10%. At the optimal projection angle and level of the target lesion, the alignment of the lesion is outlined using Stenosis Analysis. This automatically identifies the vessel edges and calculates the minimal lumen diameter of the vessel, as well as a reference diameter for the lesion. This allows the diameter stenosis of the lesion to be calculated [DS% = 1- (minimal lumen diameter/reference lumen diameter) × 100%. Vessel contours could be corrected as warranted. QCA analysis was performed by a trained analyst blinded to PVA and FFR results.

### 2.5 Physician visual assessment

All three observers (O1, O2, O3), all from our center, were composed of two Registrars and an Associate Consultant. They all have more than 7 years of experience and well experienced in interventional cardiology. They evaluated the DICOM images formed by the GE device on a screen with high resolution and interpreted the degree of stenosis on all coronary imaging independently of each other and blinded to QCA and FFR results. Follow-up studies were conducted using the mean of three observers.

### 2.6 Date analysis

The data can be divided into two main categories: continuous and categorical data. The t-student test was employed to analyze the calculations for continuous data. For categorical data, the chi-square test was employed for analysis. Pearson correlation was used to assess association between DS_PVA_ and DS_QCA_ respectively with FFR in the diagnosis of myocardial ischemia. Receiver operator characteristic (ROC) analysis was conducted to assess the performance of DS_PVA_ and DS_QCA_ in diagnosing myocardial ischemia, and the area under the ROC curve (AUC) was used as a measure of overall discriminative capability. All data was plotted using Prism10, with *P* < 0.05 representing statistical significance.

## 3 Results

### 3.1 Study population

Among the 340 patients initially screened, 293 individuals (comprising 334 blood vessels) met the study's inclusion criteria and were consequently enrolled. The characteristics of the patients and basic information were first analyzed. As details in Table 1, the mean age of the patients was 66 ± 9 years, with 82 (27.99%) of them were younger than 60 years. The mean body mass index (BMI) of the patients was 24.9 ± 11.9 kg/m^2^. The number (percentage) of male and female patients was 180 (61.43%) and 113 (38.57%), respectively. The number (percentage) of menopause women was 108 (95.58%).

**Table 1:** Characteristics of study subjects.

	Total subjects (*n* = 293)	Diffuse lesions (≥20 mm) (*n* = 227)	Focal lesions (<20 mm) (*n* = 71)	*P*-value*
Age, years	66 ± 9	66 ± 9	65 ± 10	0.4057
Age < 60, *n* (%)	82 (27.99%)	61 (26.87%)	21 (29.58%)	0.6560
BMI, kg/m^2^	24.9 ± 11.9	25.2 ± 13.5	23.9 ± 2.5	0.4489
Male, *n* (%)	180 (61.43%)	144 (63.44%)	42 (59.15%)	0.5157
Menopause of female, *n* (%)	108 (95.58%)	80 (96.38%)	27 (93.1%)	0.8302
Hypertension, *n* (%)	185 (63.14%)	152 (66.96%)	36 (50.7%)	0.0132
Hyperlipidemia, *n* (%)	58 (19.79%)	40 (17.62%)	22 (30.99%)	0.0155
Hyperglycemia, *n* (%)	81 (27.64%)	64 (28.19%)	17 (23.94%)	0.4823
Smoker, *n* (%)	102 (34.81%)	83 (36.56%)	20 (28.17%)	0.1942
Current smoker, *n* (%)	52 (17.75%)	40 (17.62%)	11 (15.49%)	0.6777

Date are presented as mean ± SD or *n* (%). BMI, body mass index.

* Between diffuse and focal.

Further examination within the paper delved into the prevalence of various risk factors among the patients: hypertension (185, 63.14%), hyperlipidemia (58, 19.79%), hyperglycemia (81, 27.64%), smoker (102, 34.81%), current smoker (past 3 months, 52, 17.75%). According to the length of stenosis, there were two groups: diffuse stenosis group (≥20 mm, 227, 77.47%) and focal stenosis group (<20 mm, 71, 24.23%). Statistical analysis revealed significant differences only in the prevalence of hypertension and hyperlipidemia between these two groups (*P* < 0.05).

### 3.2 Characteristics of lesions

A total of 334 stenotic vessels underwent both CAG and FFR assessments. The number of vessels in the diffuse stenosis group was significantly higher than that in the focal stenosis group (*P* < 0.001). As shown in the Table 2, the major locations of stenotic lesions in the vessels were the left anterior descending (LAD, 251, 75.15%), the left circumflex (LCX, 34, 10.18%), and the right coronary artery (RCA, 49,14.67%).

**Table 2:** Lesions in study subjects.

	Total lesions (*n* = 334)	Diffuse lesions (*n* = 263)	Focal lesions (*n* = 71)	*P-*value* (*P* < 0.001)
Vessel location, *n* (%)				
LAD	251 (75.15)	190 (72.24)	61 (85.91)	0.0180
LCX	34 (10.18)	31 (11.79)	3 (4.23)	0.0615
RCA	49 (14.67)	42 (15.97)	7 (9.86)	0.1966
Lesion length for the whole lesion by QCA, mm				
All vessels	30.54 ± 13.5	34.69 ± 11.98	15.18 ± 5.03	<0.001
Diameter in the lesion of interest by QCA, mm				
Reference diameter	2.85 ± 0.51	2.79 ± 0.5	3.05 ± 0.50	<0.001
Minimum diameter	1.68 ± 0.38	1.64 ± 0.37	1.83 ± 0.38	<0.001
Value of FFR				
FFR	0.87 ± 0.07	0.86 ± 0.07	0.90 ± 0.06	<0.001
FFR ≤0.8, *n* (%)	63 (18.87)	56 (21.29)	7 (9.86)	0.0289
DS of lesions				
All-DS_QCA_,%	41.27 ± 8.36	41.76 ± 8.27	39.46 ± 8.52	0.0396
<50%, *n* (%)	286 (85.62)	220 (83.65)	66 (92.95)	0.0473
50%–<70%, *n* (%)	48 (14.37)	43 (16.35)	5 (7.04)	0.0419
70%–100%, *n* (%)	0 (0)	0 (0)	0 (0)	–
All-DS_PVA_,%	57.28 ± 7.25	58.21 ± 7.2	53.85 ± 6.37	<0.001
<50%, *n* (%)	50 (14.97)	32 (12.17)	18 (5.70)	0.0057
50%–<70%, *n* (%)	268 (80.24)	216 (82.13)	52 (73.24)	0.0951
70%–100%, *n* (%)	16 (4.79)	15 (5.70)	1 (1.41)	0.1327
△DS (DS_PVA_ - DS_QCA_)	16.01 ± 7.51	16.45 ± 7.37	14.39 ± 7.83	0.0403

Date are presented as mean ± SD or *n* (%). LAD, left anterior descending; LCX, left circumflex; RCA, right coronary artery; QCA, quantitative coronary angiography; FFR, fractional flow reserve; PVA, physician visual assessment. △DS means DS_PVA_ – DS_QCA_.

* Between diffuse and focal.

The mean length of stenotic lesions was 30.54 ± 13.5 mm overall, with diffuse stenosis group exhibiting longer lesions (34.69 ± 11.98 mm) compared to the focal stenosis group (15.18 ± 5.03 mm). The reference diameter and the minimum diameter at the stenosis were 2.85 ± 0.51 mm and 1.68 ± 0.38 mm, respectively. Statistically significant differences were observed in both the reference and minimum diameters between the diffuse (2.79 ± 0.5 mm, 1.64 ± 0.37 mm) and focal (3.05 ± 0.50 mm, 1.83 ± 0.38 mm) stenosis groups (*P* < 0.001).

The overall stenosis rate was 41.27% ± 8.36%. with a greater degree of stenosis in the diffuse stenosis group (41.76% ± 8.27%) compared in the focal stenosis group (39.46% ± 8.52%) (*P* < 0.05). The degree of diameter stenosis of stenotic vessels was estimated using PVA, and the results were as follows: the overall stenotic vessels exhibited a mean diameter stenosis of 57.28% ± 7.25%. The two groups were found to be statistically significant (*P* < 0.001), with the diffuse group exhibiting a mean of 58.21% ± 7.2% and the focal group a mean of 53.85% ± 6.37%.

The FFR value of all vessel was 0.87 ± 0.07, while the FFR value of the diffuse stenosis group (0.86 ± 0.07) was smaller than that of the focal stenosis group (0.90 ± 0.06) (*P* < 0.001). Subgroup analysis revealed that 18.87% of cases had FFR values below 0.8, predominantly observed in the diffuse stenosis group (*P* < 0.05).

Coronary artery diameter stenosis is categorized according to following cut-offs: <50% (mild), 50%–<70% (moderate), 70%–100% (severe) (Table 2) (10, 11). QCA showed the largest number of stenotic vessels in the <50% (Supplementary Figure S2). The percentage of diffuse stenosis cases was higher in the <50% and 50%–70% stenosis range compared to the focal stenosis group (*P* < 0.05). PVA showed the largest number of stenotic vessels in the 50%–70%. The diffuse stenosis group (12.17%) accounted for more than the focal stenosis group (5.70%) in the 30%–50% (*P* < 0.05). The difference between PVA and QCA (ΔDS, DS_PVA_ - DS_QCA_). The mean difference was 16.01% ± 7.51% for the overall vessels, and *Δ*DS was greater in the diffuse stenosis group (16.45% ± 7.37%) than in the focal stenosis group (14.39% ± 7.83%) (*P* < 0.05).

### 3.3 Comparison of DS_QCA_ DS_PVA_ and △Ds between diffuse and focal

**Figure 1:**
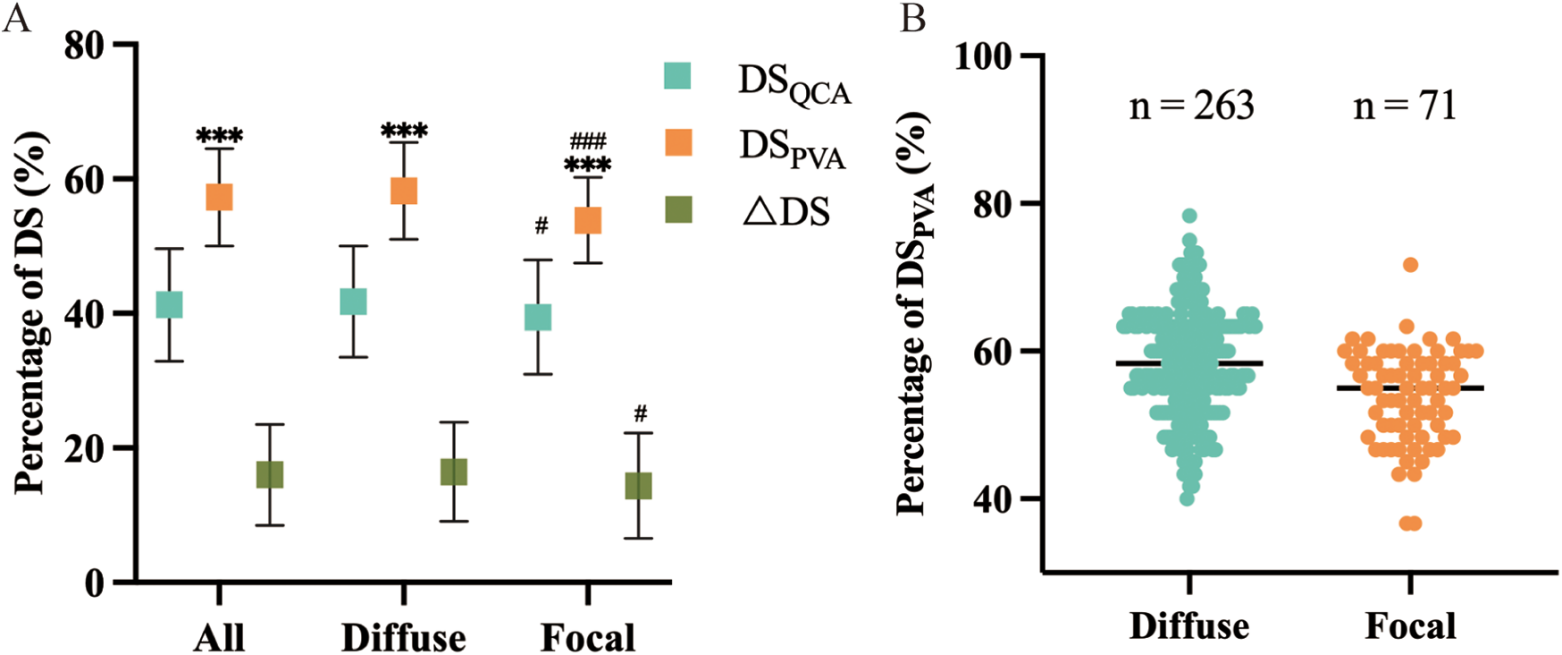
Effect of different lengths of stenotic vessels DS_QCA_ DS_PVA_ and △DS. **(A)** Comparison of within- and between-group variability for DS_QCA_ and DS_PVA_ and △DS. *means comparisons between methods within groups, ^#^means comparisons of the same method between groups, and the number of symbols represents the intensity of variability. **(B)** Distribution and frequency of DS_PVA_ measurements, and the horizontal line represents the median within groups.

In this article, QCA and PVA were employed to assess the severity of coronary artery diameter stenosis. Overall, the PVA was more likely to overestimate the degree of diameter stenosis (*P* < 0.001) (Figure 1). In the same method, the diffuse stenosis group was more likely to overestimate the degree of stenosis than the focal stenosis group (P_QCA_ < 0.05, P_PVA_ < 0.001) (Table 2; Figure 1A). The *Δ*DS was greater in the diffuse stenosis group than in the focal stenosis group. In the PVA, the median was found to be smaller in the focal stenosis group than in the diffuse stenosis group (Figure 1B). Furthermore, the focal stenosis group was observed to be relatively centralized in comparison to the diffuse stenosis group (Figure 1B).

### 3.4 Comparison of DS_QCA_ DS_PVA_ and FFR

**Figure 2:**
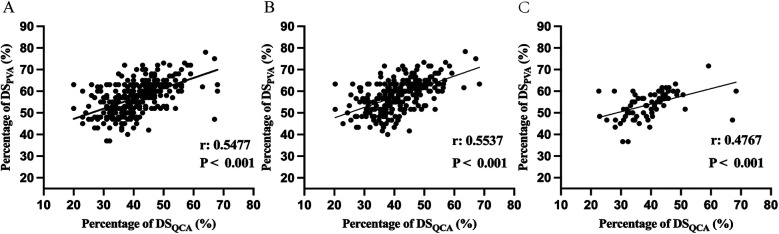
Comparison between DS_PVA_ and DS_QCA_ in **(A)** all vessel **(B)** diffuse and **(C)** focal. DS, diameter stenosis; PVA, physician visual assessment; QCA, quantitative coronary angiography.

**Figure 3:**
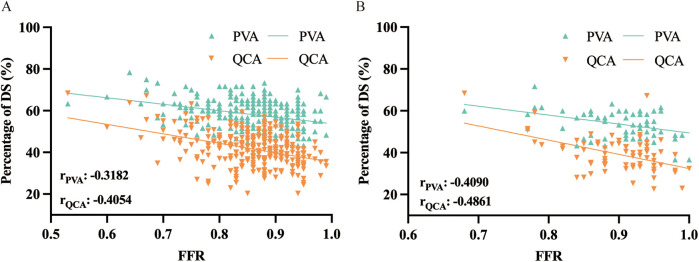
Correlation in assessment of myocardial ischemia between anatomical method of PVA and QCA and function method of FFR in **(A)** diffuse and **(B)** focal. DS, diameter stenosis; PVA, physician visual assessment; QCA, quantitative coronary angiography; FFR, fractional flow reserve.

Pearson correlation coefficient was used to analyze the association between DS_PVA_, DS_QCA_ and FFR in the diagnosis of myocardial ischemia (Figure 2A; Supplementary Figure S3). Figure 2B, C shows an assessment of DS correlation for PVA vs. QCA with a Pearson Coefficient of 0.5537 (*P* < 0.001) in diffuse and 0.4767 (*P* < 0.001) in focal, indicating a positive correlation between PVA and QCA (10). As show in the Figure 3, DS_PVA_ and DS_QCA_ had linear negative correlation (r_PVA_ = −0.3182, r_QCA_ = −0.4054) with FFR in diffuse, while in focal, DS_PVA_ and DS_QCA_ had an even stronger linear negative correlation (r_PVA_ = −0.4090, r_QCA_ = −0.4861) with FFR.

### 3.5 Diagnostic performance of DS_QCA_ DS_PVA_ in diffuse and focal

**Figure 4:**
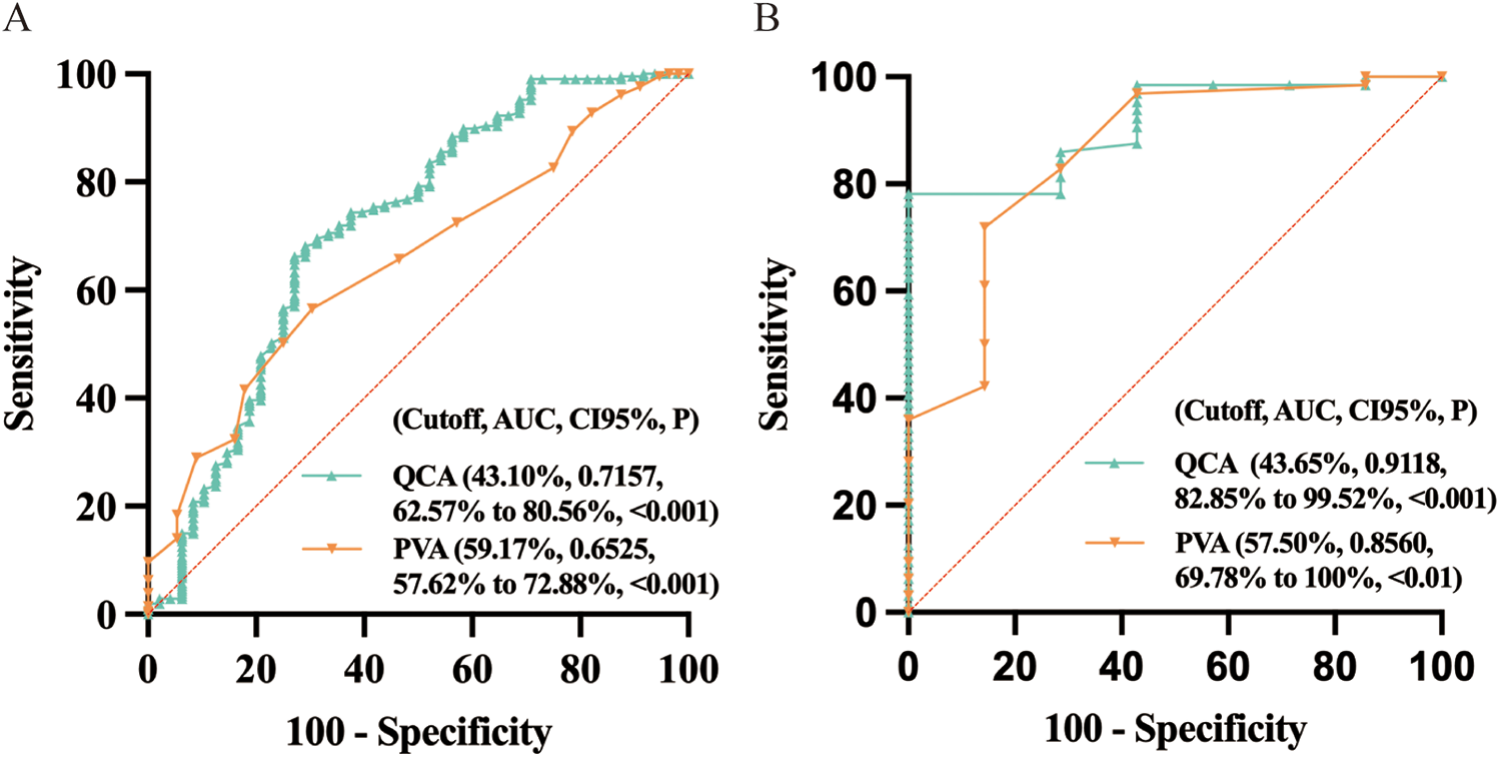
Diagnostic efficacy (FFR ≤ 0.8) of QCA and PVA. **(A)** Diffuse, **(B)** Focal. QCA, quantitative coronary angiography; PVA, physician visual assessment; FFR, fractional flow reserve; ROC, receiver operator characteristic curve; CI95%: 95% confidence interval.

ROC curves were plotted using FFR ≤ 0.8 to determine the diagnostic efficacy of DS_QCA_ and DS_PVA_. The analysis demonstrated that DS_QCA_ (AUC_QCA_ = 0.7446) was superior to DS_PVA_ (AUC_PVA_ = 0.6946) in discriminating myocardial ischemia (FFR ≤ 0.8) (Supplementary Figure S4). In the diffuse stenosis group (Figure 4A), the optimal cut-off point for QCA was 43.10% (sensitivity = 66.18%, specificity = 72.92%), with an area under the curve of 0.7157 (95% CI = 62.57% to 80.56%, *P* < 0.001). For PVA, the optimal cut-off point was 59.17% (sensitivity = 56.52%, specificity = 69.64%), with an area under the curve of 0.6525 (95% CI = 57.62% to 72.88%, *P* < 0.001). In the focal stenosis group (Figure 4B), the optimal cut-off point for QCA was 43.65% (sensitivity = 78.13%, specificity = 100%), with an area under the curve of 0.9118 (95% CI = 82.85% to 99.52%, *P* < 0.001). The optimal cut-off point for PVA was 57.50% (sensitivity = 71.88%, specificity = 85.71%), with an area under the curve of 0.8560 (95% CI = 69.78% to 100%, *P* < 0.01).

## 4 Discussion

Numerous studies have consistently highlighted coronary atherosclerosis as a predominant cause of cardiovascular-related deaths (12). Furthermore, it has been established that relatively long coronary artery stenosis is associated with more pronounced clinical symptoms and poorer therapeutic outcomes (13). The widespread use of CAG and PCI has provided new insights into the diagnosis and treatment of coronary stenosis. However, current clinical practice often relies on subjective visual assessment by the operator to determine the necessity for treatment or further evaluation of stenosis severity using techniques such as fractional flow reserve (FFR), optical coherence tomography (OCT), or intravascular ultrasound (IVUS) (14–16). While these adjunctive tests undoubtedly improve our understanding of stenosis characteristics, their intraoperative risks and economic implications cannot be ignored (17).

The disadvantages of PVA are becoming increasingly evident in clinical practice. In comparison to PVA, QCA is highly reproducible and objective. Previous reports have demonstrated that the discrepancy between observer assessment of stenosis severity by PVA and QCA ranges from 15% to 45% (9, 18–21). In this paper, a rapid and objective assessment of coronary artery stenosis using QCA was compared and analyzed with PVA. Our findings consistently showed that QCA exhibited superior correlation with the “gold standard” FFR and demonstrated higher diagnostic efficacy compared to PVA. In addition, increasing lesion length decreases the correlation and diagnostic efficacy of QCA, PVA and FFR, a phenomenon that is more evident in PVA.QCA offers a new strategy for assessing the extent of coronary stenosis by less experienced physicians and in hospitals in underdeveloped areas.

Our study identified the following issues. Firstly, it was observed that the PVA method tends to overestimate the severity of stenosis in coronary arteries (Figure 1), specifically in diffuse stenosis. This discrepancy may stem from biases such as the visual prominence of longer stenotic segments, which can create a misleading impression of severity.

The contemporary guideline recommendations lack specific guidance on treatment strategies tailored to lesion length, as they typically rely on data from studies with large sample sizes (22). Consequently, both subjective and objective factors influencing the diagnosis and management of CAD of varying lengths remain inadequately understood. The current study demonstrated that diffuse stenotic lesions exhibited a less favorable improvement in FFR and a lower treatment outcome than focal stenotic lesions following PCI (23). Furthermore, the risk of complications during the treatment of longer coronary stenoses is significantly higher (17, 24–26). Stratifying stenosis length is thus essential for providing accurate guidance in the diagnosis and treatment of CAD. To address this gap, we conducted a comparative analysis of coronary angiographic images using QCA and PVA methodologies. Our findings indicate that QCA correlates more effectively with FFR and demonstrates superior diagnostic efficacy compared to PVA. Consequently, our study contributes to enhancing the objectivity of evaluating stenosis severity in coronary images, offering a valuable alternative to subjective assessments based solely on operator experience (11). Notably, in many developing countries, reliance on PVA remains common in interventions, with the blood flow reserve fraction (FFR) being underutilized. This underscores the importance of disseminating our results to encourage the adoption of more objective evaluation methods in clinical practice.

Previous reports have similarly demonstrated that PVA is more likely to overestimate the severity of stenosis in patients with myocardial infarction than QCA (9, 27, 28).This tendency was further investigated across focal and diffuse stenoses, with our findings indicating a heightened overestimation in vessels with longer stenotic segments. Gender-specific analyses have revealed that PVA, when referenced against QCA, exhibits a greater propensity to overestimate coronary stenosis in females (10, 29). Adjedj et al. have illustrated how the presence of risk factors impacts the accuracy of both PVA and QCA assessments, with a relatively greater effect observed in PVA, particularly among diabetic patients (30). Nevertheless, none of these reports have formally analyzed lesions of different lengths (diffuse and focal).

The study presented in this paper demonstrates that QCA is a more accurate and stable method for assessing different length types of stenosis compared to PVA. This trend is particularly evident in cases of diffuse stenosis. QCA can serve as a valuable reference for young physicians or inexperienced hospitals engaged in clinical work, enabling them to optimize patient benefit. In addition, QCA has the same advantages as iFR in that it does not require injections of vasodilators (adenosine or ATP) when compared to expensive FFR procedures (31). In addition to this, QCA also has the advantage of being comparable to quantitative flow ratios (QFR). Both QCA and QFR can be employed for offline diagnostic evaluation of coronary stenosis based on XA images, obviating the need for drug injection or additional guide wires (32). While reducing costs, intraoperative risks are also significantly reduced.

## 5 Limitations

There are some limitations to this study. First, our date come from a tertiary grade “A” class hospital-the best tertiary hospitals are defined as those categorized as tertiary and graded “A”. This may have resulted in a general underestimation of the disparity in the assessment of diameter stenosis between PVA and QCA, when compared to what would be observed in lower-grade hospitals (“B” and “C”) with potentially outdated coronary angiography technology and lower levels of physician proficiency in PVA evaluation and clinical interpretation. Secondly, this was a retrospective, single-centre study and the patients were mainly from the south-western region of China, which may not be representative of the whole region. Subsequently, this study lacks the actual impact of QCA on clinical decision-making and patient outcomes. Finally, the number of focal stenoses in the patient sample was relatively small, which may introduce some error.

## 6 Conclusions

In this study, PVA was more likely to overestimate diameter stenosis in coronary arteries than QCA, and the severity of diffuse stenosis was more likely to be overestimated than that of focal stenosis. This article recommends the incorporation of QCA into clinical practice, which would ensure more accurate interpretation of coronary angiography and inform optimal therapeutic decisions in longer stenotic coronary vessels.

## Data Availability

The original contributions presented in the study are included in the article/Supplementary Material, further inquiries can be directed to the corresponding authors.
