# Supplementary material for: Differences in severity of diffuse and focal coronary stenosis between visual and quantitative assessment
Source: Front Cardiovasc Med. 2024 Dec 16;11:1501576. doi: 10.3389/fcvm.2024.1501576 (PMC11683054; doi:10.3389/fcvm.2024.1501576)
Supplement: Supplementary file 1 [file Datasheet1.pdf]

Supplementary Material

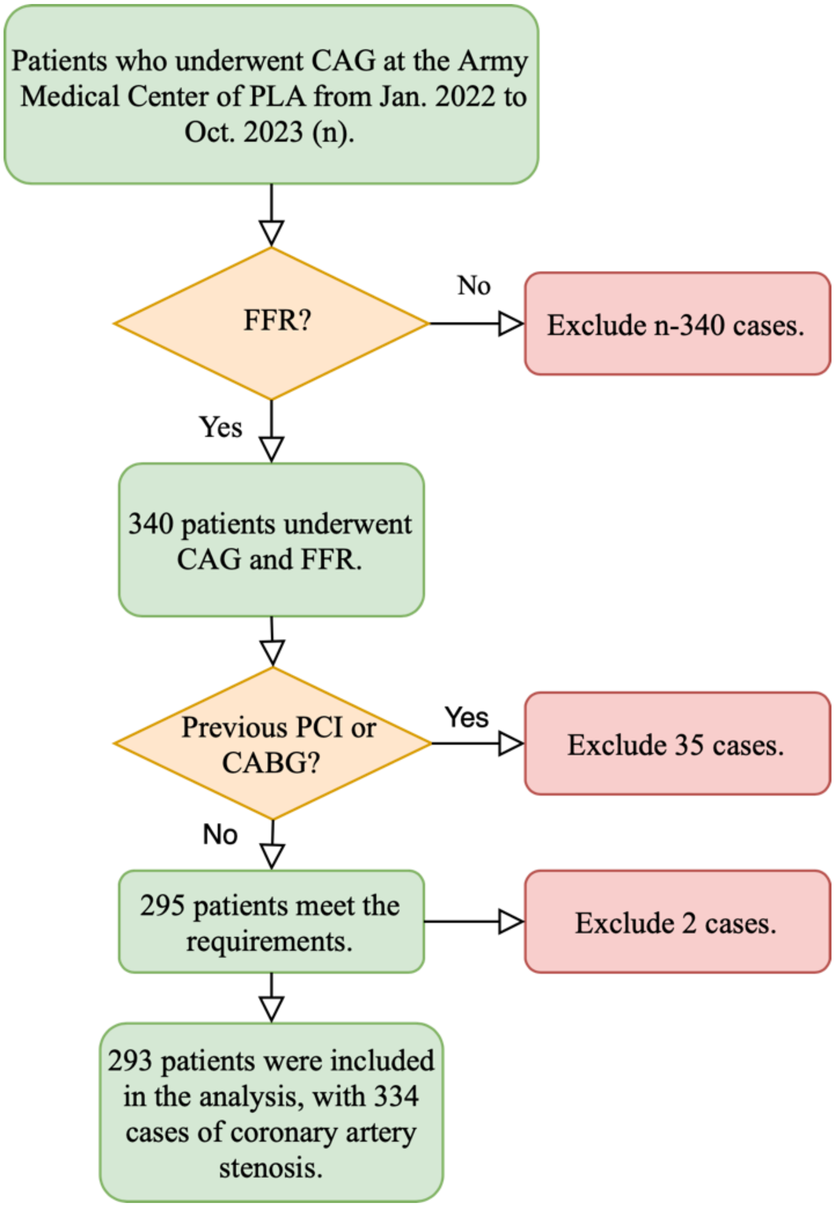

Figure S1. Requirements for case enrolment and exclusion criteria.

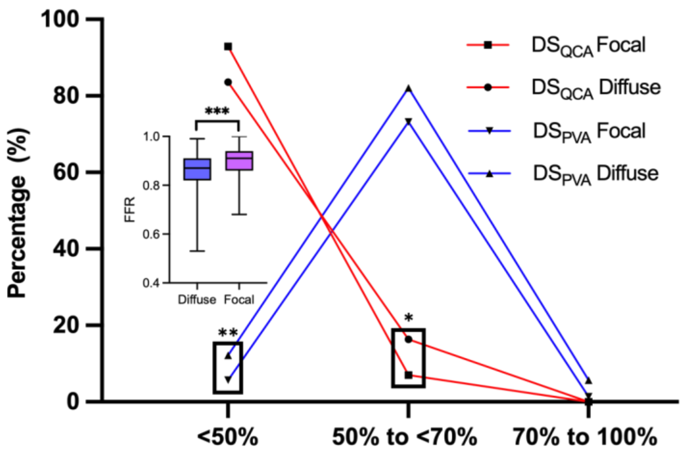

Figure S2. Distribution of severity of diffuse and focal stenosis vessels using both QCA and PVA, with inset plots of FFR in diffuse and focal stenosis.

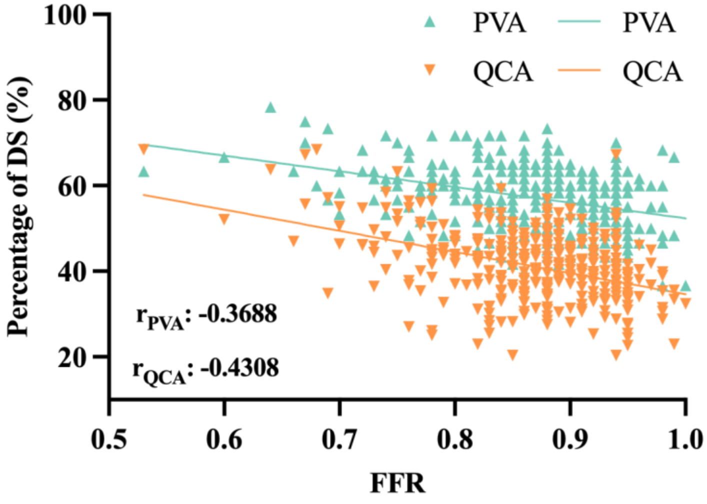

Figure S3. Correlation in assessment of myocardial ischemia between anatomical method of PVA and QCA and function method of FFR in all vessels.

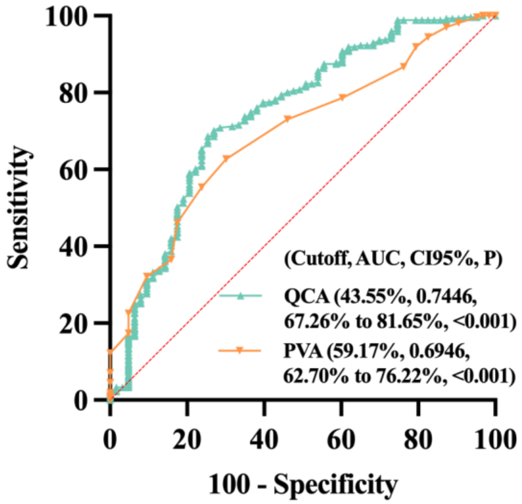

Figure S4. Diagnostic efficacy (FFR< 0.8) of QCA and PVA.
